# Assessment of function and clinical utility of alcohol and other drug web sites: An observational, qualitative study

**DOI:** 10.1186/1471-2458-11-277

**Published:** 2011-05-05

**Authors:** Frances J Kay-Lambkin, Angela White, Amanda L Baker, David J Kavanagh, Britt Klein, Judith Proudfoot, Judy Drennan, Jason Connor, Ross M Young

**Affiliations:** 1 National Drug and Alcohol Research Centre, University of New South Wales, Australia; 2 Centre for Brain and Mental Health Research, Faculty of Health, University of Newcastle, New South Wales, Australia; 3 Centre for Youth Substance Abuse Research, Faculty of Health Sciences, University of Queensland, Australia; 4 Institute of Health & Biomedical Innovation and School of Psychology & Counselling, Queensland University of Technology, Queensland, Australia; 5 National eTherapy Centre, Faculty of Life and Social Sciences, Swinburne University, Victoria, Australia; 6 BlackDog Institute, School of Psychiatry, University of New South Wales, New South Wales, Australia; 7 School of Advertising, Marketing and Public Relations, Faculty of Business, Queensland University of Technology, Brisbane, Queensland, Australia

## Abstract

**Background:**

The increasing popularity and use of the internet makes it an attractive option for providing health information and treatment, including alcohol/other drug use. There is limited research examining how people identify and access information about alcohol or other drug (AOD) use online, or how they assess the usefulness of the information presented. This study examined the strategies that individuals used to identify and navigate a range of AOD websites, along with the attitudes concerning presentation and content.

**Methods:**

Members of the general community in Brisbane and Roma (Queensland, Australia) were invited to participate in a 30-minute search of the internet for sites related to AOD use, followed by a focus group discussion. Fifty one subjects participated in the study across nine focus groups.

**Results:**

Participants spent a maximum of 6.5 minutes on any one website, and less if the user was under 25 years of age. Time spent was as little as 2 minutes if the website was not the first accessed. Participants recommended that AOD-related websites should have an engaging home or index page, which quickly and accurately portrayed the site's objectives, and provided clear site navigation options. Website content should clearly match the title and description of the site that is used by internet search engines. Participants supported the development of a portal for AOD websites, suggesting that it would greatly facilitate access and navigation.

Treatment programs delivered online were initially viewed with caution. This appeared to be due to limited understanding of what constituted online treatment, including its potential efficacy.

**Conclusions:**

A range of recommendations arise from this study regarding the design and development of websites, particularly those related to AOD use. These include prudent use of text and information on any one webpage, the use of graphics and colours, and clear, uncluttered navigation options. Implications for future website development are discussed.

## Background

The rise in popularity and capability of the internet has led to a revolution in the provision of health-related information and treatment. Over one-quarter of the world's population currently have internet access [1], and over 75% of internet users have sought health-related information online [2].

Internationally, substantial numbers of people report problematic alcohol and other drug (AOD) use. The incidence of substance use disorders (excluding nicotine use) in the general population is approximately 9% [3], making it one of the most common mental health problems in western society [4]. Add to this the negative impact such disorders can have upon physical health, psychological status and social functioning [5], along with the prediction that by 2020 the global burden of disease attributable to alcohol and illicit drug use will be in the range of 30,962,000 lives [6], substance use represents a significant public health issue. Receipt of treatment by people with AOD problems falls far short of treatment needs, particularly for counselling interventions [7]. Alternative cost-effective strategies to improve treatment access are required.

Internet access is common among people with AOD use problems. A Canadian survey reported that current drinkers were significantly more likely than abstainers to have internet access at home (73% vs. 50%), with illicit drug users (e.g. cannabis and cocaine) reporting equivalent rates of home internet access to non-users [8]. Descriptive research indicates that people with AOD problems find internet-delivered screening and treatment acceptable [9]. Several studies conducted among people with hazardous alcohol use indicate that this group access online AOD material at rates would overwhelm traditional drug and alcohol services if translated into occasions of service [e.g. average of 34 visits per day, 10]. Internet-based treatments for AOD use may have particular appeal to people who cannot access traditional services due to geographical, financial or attitudinal barriers [11]. The 24-hour availability of online programs and resources is an important benefit that existing face to face services are unable to match [12]. Indeed, the out-of-hours availability along with anonymity and privacy afforded by the medium, are frequently cited reason for individuals use of online AOD resources and materials [10,13].

However, very little published data are available to indicate ways in which internet-delivered information and treatment should be developed and presented, in order to capitalize on these potential benefits [14]. Despite a recent surge in websites providing AOD-related information [15], we also do not know how well existing sites are able to engage site users, to meet their needs or expectations. Having access to this information will enable website developers and treatment providers to maximize the impact of existing AOD websites and provide guidance for how to improve on this in the future.

This paper reports on direct observation of online use of AOD websites by members of the general community, and results of subsequent focus group discussions. This study aimed to determine the methods people used to identify and access AOD websites, and observe how long they stayed on a site, when asked to evaluate it. The study also assessed their expectations and preferences for existing AOD websites, and their suggestions for marketing and promotion of AOD-related information online. The current study represents the first to employ qualitative methods in examining the attitudes of a sample of the general community towards AOD-related websites.

## Methods

### Participants

Members of the general community in rural and urban Queensland, Australia, aged 16-25 (n = 20), and 26-65 (n = 17) were recruited to the study, as were participants from Aboriginal and/or Torres Strait Islander (ATSI, n = 9) and culturally and linguistically diverse backgrounds (CALD, n = 5). Participants were recruited through locally distributed flyers and promotion through local metropolitan and regional radio and newspaper outlets. Study participants were offered $50AUD reimbursement for time and travel expenses associated with participation (those aged 16-18 years received a $50 shopping voucher). Ethics approval was granted via the Human Research Ethics Committee, University of Queensland, Australia (Approval number: 0900000114), and the study took place January-June 2010.

### Measures

A participant log and focus group protocol were developed to track participant progress and basic impressions of the AOD sites visited. An online survey developed for the general public to evaluate drug and alcohol internet services [16] was used to collect basic demographic information from participants.

### Procedure

A series of four rural and five metropolitan focus groups were conducted, involving 51 participants, who consecutively volunteered for the study in response to project advertisements. Data collection took place at the Queensland University of Technology (Brisbane), or at the local community education centre in Roma.

### Unguided internet search

Participants were initially asked to complete a search of the internet for AOD-related websites, generating their own search terms and strategies. During this phase (approximately 30 minutes), participants kept a register of their activities, websites visited, time spent on each website, and associated impressions, and were asked to review and evaluate up to five AOD related websites.

### Focus group discussion

A one-hour group discussion followed. Using a standardized set of open ended questions, participants were asked to comment on the websites they visited, as per Table 1. The same facilitators led all focus groups (AW + research assistant), and groups comprised 6-8 members each. Focus group discussions were videotaped for transcription and analysis.

**Table 1:** Focus group stimulus questions

General Theme	Stimulus question
**Website Evaluation**	*"Think about the websites you found in your free search of the Internet. What made some sites better than others? " *
**Gaps**	*"Of the AOD websites you visited, was there anything you wanted to find, but couldn't? Did the websites generally meet your expectations, or were there things missing that you feel should have been there?"*
**Online Treatment**	*"After having a look at these websites, what do you think about online treatment for alcohol/other drug problems?"*
**Promotion/dissemination**	*"Do you have any ideas about promoting AOD websites to people like yourself?"*

Analysis of transcripts took a thematic analysis approach [17], with the study team (FKL, AW, AB) identifying the main themes emerging from each discussion and highlighting relevant verbatim quotes that represented each main theme. To maximize the reliability of this phase, all focus group transcripts were reviewed in this manner by two groups within the study team (FKL+AB, AW). Any discrepancies were discussed and resolved consensually. When reporting results below, illustrative quotations are presented. Editorial omissions to these quotations are indicated (...).

### Online questionnaire

As a final step, focus group participants provided demographic and internet-usage data via an online survey. The online survey has been described in detail previously [18], and asked respondents about a number of areas of their typical internet useage and experience including:

• General internet useage;

• AOD website internet use and behaviors;

• Most visited AOD websites;

• General website features;

• Interactive website features;

• Judgin website trustworthiness;

• Preferences regarding AOD website tools/functions and online treatment.

Forty-three participants completed the online survey (84% of all focus group participants), with the remainder opting out of this phase of data collection due to time constraints.

## Results

Fifty-four percent (n = 23) of our sample was female, 7% (n = 3) identified an ATSI background, and 21% (n = 9) reported that English was not their first language. One-third of participants (n = 15) were currently single, and 33% (n = 14) were currently living with a partner. Most were employed at least part-time (n = 25, 58%), with 16% (n = 7) receiving disability or unemployment benefits. The majority of respondents had completed secondary schooling (n = 27, 63%).

### General internet usage

Of the participants completing the online survey, 31 (72%) said their primary means of accessing the internet was at home, either via a computer with cable broadband internet access (n = 18), or ADSL internet access (n = 9).

Most respondents (n = 35, 81%) accessed the internet on a daily basis. Most commonly, they spent was an average of 30-60 minutes online each time (n = 16, 37%; Range = 10-30 minutes to more than 3 hours). Most (n = 37, 86%) felt comfortable and confident in using the internet.

### Internet usage for health-related concerns

Eighty-one percent had previously accessed the internet for health concerns, or health-related information. This access was most commonly for information about physical health and illness (n = 26, 61%). Information about alcohol was sought by 37%, and information about drugs by 47%. Access to information about health-related issues was typically from respondents' home computers (n = 28, 80%), or from their work, school or University terminals (n = 16, 46%). Few (n = 2) accessed the internet in public locations to search for this type of information.

### Internet usage for AOD-related concerns

Participants indicated that, of the AOD-related websites they had accessed in the past, the focus was mainly on drug-related issues (n = 19, 42%) or those that dealt with alcohol-related issues (n = 10, 23%). Fifteen respondents (35%) had never previously visited a website that addressed AOD issues.

When accessing AOD-related websites in the past, respondents were generally looking for information about substance effects (Alcohol: 78%, Other drugs: 83%), about supporting control attempts by substance users (Alcohol: 67%, Other drugs: 47%), or about support for family members (Alcohol: 63%, Other drugs: 41%).

### Observation of the Unguided internet search

With two exceptions, participants used Google for their free search. Their most common search terms were: "drugs and alcohol" (n = 13) or "drug and alcohol use" (n = 5), which were combined with terms such as "abuse" (n = 7), "addiction" (n = 3), "effects" (n = 2), "help" (n = 3) "issues" (n = 2) or "facts" (n = 2). Some participants focused on alcohol, using terms such as "alcoholism" (n = 1), "alcohol" (n = 4) ord "alcohol misuse" (n = 1), while others focussed on "prevention" (n = 1), "information" (n = 1), "drugs" (n = 1), "caffeine" (n = 1) or "foetal alcohol syndrome" (n = 1). Ten specified "Australian sites only" in their search strategy. In most cases, the initial search strategy was the sole one used in the period of free search.

Once a website had been identified, the average time spent on any website during the 30 minute free search phase ranged from 2:48 minutes (16-25 year group) to 6:36 minutes (CALD group). The time spent exploring a website decreased as the free search period went on, and the 16-25 year group typically spent the least time on each website (see Table 2).

**Table 2:** Proportion of focus group participants, according to age group or cultural background, indicating their reasons for accessing websites related to AOD use.

Domain	16-24 years	25-65 years	CALD^a^	ATSI^b^
**Average time spent**				
1^st ^website	4:18 mins	5:30 mins	6:36 mins	6:13 mins
2^nd ^website	4:00 mins	5:30 mins	6:00 mins	5:00 mins
3^rd ^website	3:12 mins	4:18 mins	5:30 mins	5:24 mins
4^th ^website	2:48 mins	2:42 mins	6:15 mins	3:42 mins
5^th ^website	3:24 mins	2:24 mins	3:30 mins	-
**Top reasons for selecting website^c^**				
First on list	25% (n = 5)	24% (n = 4)	20% (n = 1)	22% (n = 2)
Government source	55% (n = 11)	18% (n = 3)	**80% (n = 4)**	0% (n = 0)
Source looked reliable	45% (n = 9)	29% (n = 5)	0% (n = 0)	22% (n = 2)
Title clearly matched search terms	**75% (n = 15)**	12% (n = 2)	20% (n = 1)	33% (n = 3)
Interesting title and description	65% (n = 13)	29% (n = 5)	20% (n = 1)	11% (n = 1)
Potential information focus	20% (n = 4)	**59% (n = 10)**	0% (n = 0)	**67% (n = 6)**
Potential treatment focus	20% (n = 5)	41% (n = 7)	20% (n = 1)	33% (n = 3)

^a^People from culturally and linguistically diverse backgrounds.

^b^People from Aboriginal and/or Torres Strait Islander backgrounds. No members of the ATSI group accessed a fifth website.

^c^Most common reasons are in bold.

Participants commonly chose the first website on the list generated by the search engine to explore first (16-25 year group: 55%, 25-65 year group: 59%, ATSI group: 44%, CALD group: 40%). However, when subsequently asked, the main reason for choosing a site to access first, the order of sites in search results was only cited by about 20-25% of participants (Table 2).

### Focus group discussion

#### Website evaluation

Participants were asked to comment on features of websites they visited during the unguided search, which contributed to their ease of use and appeal. The main issues to emerge were: effective information provision; objective, non-judgemental approaches; defined/limited to a specific target audience; interactivity that personalised the website access; clear, simple layout and design; attractive, aesthetically pleasing characteristics; relevant life experiences or graphics; a match between the website's content and the title and description of the site provided by the search engine; and whether it was provided by known and trusted organisations.

Across focus groups, websites that provided clear, concise information regarding AOD use were considered the best. Key features of sites that did this best were use of images and pictures in place of text, provision of evidence to support information, avoidance of jargon (e.g. used street names of drugs), and presentation of information that was easily understood and accessible by a range of people who differ in reading ability, internet use history and cultural background.

*"Plenty of them had too much information... it takes a lot of time, and whoever has the problems, I assume, doesn't have enough patience..." *[Member of CALD Group]

Participants in the 16-25 year focus groups said the tone of language was critical in their perception of usefulness and acceptability. Websites that seemed objective, unbiased and non-judgemental were more acceptable than ones that took a *"preachy" *approach to information provision. Explanations of why and how issues were relevant and important to the issue of AOD use were valued, as opposed to provision of advice about what *"should" *or *"shouldn't" *be done.

*"...to me I think, when these websites say don't do this, don't do that, I don't want to listen...whereas if it says if you are going to do it, do it responsibly... that is more interesting." *[Member of 16-25 year groups]

Clear website affiliations were not as important for younger people (16-25 group) than older participants, nor was having knowledge about who created and maintained the website.

*"I'd look for their policy statement or something, and their mission statement, anything that gives a sense of their values, and I tend to look for it, to be honest. I tend to have in my mind, I won't go further if this doesn't tell me where they are coming from..." *[Member from 25-65 year Groups]

Websites that were tailored to a specific audience, and only provided information that was relevant to that audience (e.g. young people), were also valued. This included the layout and pitching of website content, and linkage to geographically-relevant information and resources to the user's city or rural location).

Greater interactive options increased a website's appeal to younger participants. Opportunities to complete self-assessments online (with caveats regarding accuracy and validity of results), quizzes, question and answer sections and animations were all identified as features of a website that kept attention of site users. Interactivity options needed to be relevant to the site's target group, and needed to engage users while not distracting attention away from the seriousness of the issues addressed.

All focus group discussions considered the layout of the website (and particularly the home page) of paramount importance in rendering it appealing, engaging, interesting and accessible. Key features were clear, easy-to-read text (including level of language and style of font), a *"clean"*, uncluttered layout (avoiding excessive text, graphics), simple navigation (including ability to readily returning to the home page), and a logical flow of information throughout the site. A site's *"look and feel" *was important to many participants. Minimizing "pop-up" advertising and other promotional information reportedly affected perceptions of 25-65 year participants. Websites needed to be *"eye-catching within the first few seconds"*, with engaging use of colour, graphics or images and interactive options.

*"I like colour- believe it or not, and I just feel, when it is very clinically written, I just sit back. But when it is colour and it gives a genuine feeling of welcome, then I wanted to have a look..." *[Member of 25-65 year Groups]

Participants suggested that links to other websites and resources should be minimised, and information relevant to the website's objectives (and to the target audience) should be accessible and contained within the website.

*"...you don't want to have to go through too many links either...you just want *[it] *to come up on the page...for it to be clear, concise and to the point, and not having to filter through different links... it gets confusing..." *[Member from 16-25 year Groups]

Some focus group members in the 16-25 year, 25-65 year and the ATSI groups said that inclusion of life stories was important, as it personalised the experience (and accentuated the relevance) of information. Young people suggested that including success stories available, such as the *"rise and fall and rise again" *of well-known personalities may engender hope. Younger participants also suggested that inclusion of graphic images of the consequences of AOD use added a curiosity factor to some websites, encouraging users to explore the website further.

Crucial to a website's appeal was that expectations for its content were met. The title of a website (or the URL) and the description provided by the search engine needed to match the objectives of the site and what it offered. A title that minimised use of jargon was more appealing, as was a *"catchy" *name, as it would be easily remembered and recognised. Ranking on the search engine list was also considered important.

A few participants felt most comfortable visiting websites that represented organisations with a public profile separate from the internet. Well-known (and therefore trusted) community groups or companies were frequently visited by participants, as were sites with ".org" or ".gov" in their address. Federal and state-sponsored websites were often the first port of call and were considered a safe place to start a search for sensitive information.

*"I was pretty new in this kind of internet researching and, I wanted to get into some very general first sites, first choices were government, and I spent more time in a few of them, 'cause I wanted to see all of the things that they are offering there...' *[Member of CALD group]

#### Gaps in existing online resources for AOD use

When asked about what was missing in their online search results, participants identified the following: websites related to prevention; websites related to comorbidity; explanations as to why AOD use can be harmful; practical advice and suggestions; a "hub" site or AOD portal through which other websites can be accessed easily; graphics of illicit drugs and consequences of use; online forums; the ability to access immediate help; websites containing simple information and that used images; and assistance for non-English speaking persons.

Young people wanted practical advice and suggestions about **how **to stop drinking or bingeing, rather than just advice to avoid this alcohol use. They suggested that information and advice to families and friends of AOD users was not available online, with many websites instead providing links to telephone advice.

*"...I know at the end of the day you need to know what those issues are, but there seemed to be too much of a focus on that, rather than how to help people or where you can refer too..." *[Member from 16-25 year Group]

A harm reduction message was sought by young people with practical tips about how to minimise or avoid risks.

*"I guess how to like, you can't stop drinking all of a sudden, there was nothing that said how to ease off, it was just, don't do it all. Or you will go to jail, blah, blah, blah...I just switched off" *[Member from 16-25 year Groups]

Participants across the focus groups wanted a hub or portal site that provided a *"one stop shop" *for AOD information. That site would contain links relating to AOD issues e (e.g. information about drugs, how to assist friends, legal information, etc.) and would be placed high on search engine results. This approach was also a way to access local information.

*"...as far as the information is concerned, in the website stuff, a uniform *[approach] *would be good, so that everyone can access the same information in the same ways..."*. [Member from CALD Group]

There was a lack of websites providing concise information in plain English that was coupled with relevant images. This type of information was identified as being particularly important to people with limited facility in written English. No website seemed to accommodate people from CALD backgrounds.

Older participants (25+ groups) and those from CALD groups found it surprising they could not access immediate help online.

*"You asked did the website meet our expectations... I thought if someone was trying to get help, from the internet, how to solve a problem, the first thing I expected is, online counselling or something like that, so you don't go to GP, you don't go nowhere, you just want to get help *[online]... *I found it on none of the websites..." *[Member from CALD Group]

#### Acceptability of online treatment for AOD use

The most common response when asked for opinions about online AOD treatment was confusion. In every group, the facilitator spent several minutes providing examples of potential online treatments. This appeared largely due to a lack of previous experience with online AOD treatment programs.

*"Like, how so?" *[Member from 16-25 year Groups]

*"You mean like online AA?" *[Member from 16-25 year Groups]

*"...do you mean like...an online messenger thing, were you could chat with, I guess, someone at the other end?" *[Member from 16-25 year Groups]

*"How do you talk to someone on the internet, unless you are like chatting on msn? Is that just giving information?" [*Member from 16-25 year Groups]

*"So you are saying, what do you mean by treatment, do you mean abstaining for a period of time or...?" *[Member from 25-65 year Groups]

Initial responses of each group towards the idea of online treatment were cautious and somewhat negative, with concerns raised regarding the perceived impersonal nature of online treatment, reliability and validity of reports of behaviour and of suggested strategies, and concern about the degree of access to ongoing maintenance and support.

As discussion developed, participants became more open to the idea of accessing online treatment, but saw it as being dependent on characteristics of the person seeking treatment, as an initial step in the treatment process, and as primarily relevant to situations where confidentiality and anonymity were especially important.

Concerns were raised by each group regarding the impersonal nature of online treatment, and the perception that internet-based treatment was a *"one size fits all" *approach to addressing a very individual problem. Participants said that, particularly for AOD related issues, face-to-face contact was important in order to *"make treatment real" *and to encourage people in therapy to take full responsibility for their problems.

Internet-based treatment was also seen to be *"easier to ignore"*. Concerns were raised that less commitment to treatment was likely, as there were fewer opportunities for engagement with a therapist and treatment program. It would be easier to disengage or *"not to bother and drop out" *from online programs than from face-to-face treatment.

The reliability and validity of treatment online was brought into question by many participants across the focus groups. Participants were anxious about the potential for people to misrepresent themselves as therapists online, without an accurate way to determine a therapist's qualifications, or their true identity.

Some participants felt that dangers of online self-diagnosis and treatment outweighed its benefits, raising concerns raised about information, treatment strategies and advice being misinterpreted and misused by site users.

*"Text can be provided in different interpretations, to different people, so *[the way they] *might interpret it is different from somebody else...the information might then not turn out to be accurate, and mightn't be helpful..." *[Member from 16-25 year Groups]

Other participants strongly argued that online treatment sites should be evaluated to ensure that they worked in the intended way.

After considering a range of possibilities for online treatment provision, participants were generally supportive of the idea. Depending on the individual and the circumstances, this mode of delivery may have important applications.

*"I think it could be interesting, 'cause you are not going to freak out and stuff, and you can just try, just try at home, you know? Get an idea you know." *[Member from 16-25 year Groups]

Participants suggested that online treatment would require more motivation and commitment than would be required from face-to-face approaches. Individuals pursuing online treatment would need this to be explained upfront.

Internet-delivered treatment was seen as a potentially useful initial step within a larger therapeutic process, rather than being suitable for more severe problems.

*"But maybe with a screening process, like if you thought you had an addiction and you were trying to break it, you jump on and you go, I smoke marijuana three times a day, what can I do, they will say well maybe go and see your GP or well find out some information, and maybe build up a bit of a profile and help them to access treatment." *[Member from 16-25 year Groups]

Participants said that websites would be good for online screening, provision of brief advice (including links to appropriate support services) for people with mild to moderate problems, and for online contact with another person (a therapist or sponsor) to whom the site user is accountable for change and program completion.

For people with concerns about privacy and confidentiality, the internet-based approach was seen as potentially allowing access to resources in a safe and secure environment.

"... some people wouldn't be able to walk into a doctor's, and you know and tell your parents, it is like nope...It is easier to talk to a complete stranger and type it in..."

[Member from 16-25 year Groups]

*"I just had a thought, you are from a small town ...there is a privacy thing, everyone knows everyone, if you are in the waiting room, somewhere, you really don't want someone to see you in a waiting room somewhere... it is the privacy thing of the internet, you know at least getting some of the idea, getting started..." *[Member from 25-65 year Groups]

#### Ideas for promotion and dissemination of websites related to AOD use

Participants had a range of ideas for marketing of AOD websites, including: a portal or hub site that could be branded and promoted, advertising the websites on other sites, and, the use of interactivity sites such as Facebook and Twitter.

Specific suggestions included paid television advertisements and print media, advertising on the side of buses, graphic images or slogans on alcohol bottles, merchandising website logos (e.g. on stickers, hats, magnets, t-shirts), sponsored links on search engines, promotion in bars, or community events, or using word of mouth.

*"like all the forums, all the messenger stuff, like you need *[something] *that they can forward on to all their friends, cause it is all about forwarding...*

*"but within that information, you need to have something relevant, not just have, not just don't use drugs..." *[Members from 16-25 year Groups]

Some participants suggested promoting websites through schools, developing "catchy" titles for sites and branding to facilitate website recognition, placing promotional material in General Practice waiting rooms, recruiting a well known person for an advertising campaign, using reality television approaches to describe personal journeys involving AOD use, and using organisations that are known to the target group.

To continue the engagement of registered site users, they suggested that SMSs be sent to their mobile phones at key times when AOD use was likely (e.g. early to late evening on weekends),

"...you could actually set up something before you went out, to send yourself an SMS text ... to remind yourself to eat between drinks, or alternate your drinks, or ... when the last train or bus was leaving to get home safely."

[Member from 16-25 year Groups]

## Discussion

This project sought to explore the experiences and impressions of a sample of community members in identifying and using websites providing AOD-related information. The key observations arising from this study are related to how the sample identified and selected AOD websites to visit, recommendations regarding the content, presentation and navigation of AOD websites, and preferences for how the internet might be used for AOD-related concerns.

Minimising use of text-based pages, presenting few key messages on each page, making good use of colour, representing information graphically, and having clear, logical site navigation were key issues for all groups, regardless of age or cultural background. The tone of language was also important, with information and advice needing to display a harm reduction approach and non-judgemental attitude if it were to have a substantial impact.

During free search of the internet, participants only stayed on a site for an average of 6.5 minutes at most; less if the user was under 25 years old, and for just 2 minutes if the site was not the first one accessed. This result suggests that AOD website developers use an engaging home page that quickly and accurately portrays the site's key objectives, and provides clear navigation options for accessing other areas in the site. Website content should match the title and description that is used by the search engine to encourage access. These issues emerged in all phases of data collection and across all age and cultural groupings, and has been supported in similar research conducted with adolescents regarding internet-delivered behaviour change interventions [14].

Participants were initially unenthusiastic about obtaining treatment via the internet, especially if there was no support person involved. In addition, focus group data indicated that participants had not encountered online treatment previously, and struggled to imagine how it would work. Ignorance of online treatment and biases against it may represent a major barrier to its widespread uptake of internet-based treatment, which may well outweigh its potential benefits of accessibility, confidentiality, immediacy, and cost-effectiveness [19]. While attitudes of the current general community sample may not reflect the views of people with AOD use problems the challenge for providers of online AOD treatment is to raise the profile of its potential benefit. Once this was done in the focus group discussions, participants agreed that online treatment could be useful screening and initial intervention, and where confidentiality and anonymity were of paramount importance.

Several gaps were identified by participants during their internet search. Participants did not identify any Australian websites for people from CALD backgrounds, with participants resorting to sites geared towards Indigenous people, on the rationale that issues relevant for this population might also apply to them. Participants could not find websites in languages other than English, and suggested that providing information on how to access interpreter services and a greater use of images and simpler language on existing websites would facilitate comprehension. CALD participants particularly found government-sponsored sites trustworthy, highlighting a potential opportunity for suitably minded governments to address this important gap in internet service provision and information. This approach may also benefit people of ATSI backgrounds, especially if the site is geared toward the provision of information rather than treatment.

Websites related to prevention and to comorbidity were not found by focus group participants, and were identified as being important and relevant. Online treatment was not easy to find, and although initially considered not ideal, participants in the focus group discussion felt that websites explaining why AOD use can be harmful, and offering practical advice and suggestions to users, friends and parents would be useful. Participants also recommended the development of a portal website that provided links to AOD websites that were certified for quality and currency. Given that focus group participants highly valued government-endorsed or sponsored sites as being reliable, current, and trustworthy, a government-sponsored portal website may be most appropriate.

Limitations to the study include its restriction to Australian residents, recruited from the community to a study examining AOD-related internet usage. We do not know how well the results may generalise to other contexts. Relative to the Australian population, our sample had equivalent rates of females (54% vs 51%), were younger on average (31 years vs 37 years), and had lower rates of employment [58% vs 65%, 20]. Our sample reported higher rates of high school completion than the general population of Australia (63% vs 21%), and lower rates of co-habitation [33% vs 50%, 20]. However, the sample included higher proportions of people of Aboriginal and/or Torres Strait Islander descent (7% vs 2.5%) and of people from culturally and linguistically diverse backgrounds (21% vs 14%). Generally in Australia, internet use is affected by age (higher use amongst younger age groups), education, and income, with significantly lower rates of access among people of Aboriginal and/or Torres Strait Islander backgrounds [36% vs 72%, 21]. In Australia, 72% of households report home access to the internet [21]; an identical rate to that reported by participants in our sample. Importantly, although 81% of the sample accessed the internet on a daily basis, 35% had not previously used the internet to explore information related to AOD. Taken together, this information suggests that our sample may provide some indications of responses that may be seen in a larger, representative sample. We also do not know the AOD backgrounds of our sample and therefore how they may relate to people with AOD use problems seeking to use the internet for support for their problems. Finally, statements about relative preferences of younger or older participants and of ATSI or CALD samples should be treated with particular caution, given the very small sizes of these sub-samples.

A final consideration is the issue of website maintenance and currency, which was not addressed specifically by participants in this study, but is implicated in the recommendations of the groups. It is costly to set up and maintain an active website that includes interactive components, collects screening data, provides interpretation of screening results, and interactive treatment programs delivered and supported online. It is of further cost to imbed and maintain appropriate security protocols to protect the privacy and confidentiality of site users. In order to maintain site currency and interest, it will be necessary to update information (as new research and policy emerges), update treatment and screening tools (as the evidence base expands), and to modify existing sites as new technology develops and along with it, site user expectations. Any website development team needs to be mindful of this, and committed to ongoing maintenance and development of a website beyond the initial setup and activation. Guidelines need to be developed that assist website development teams to incorporate evidence based practices into any website, particularly related to AOD use, and that can also assist site users to quickly and accurately determine the trustworthiness of any given website.

## Conclusions

This study provides important information about potential features of existing AOD websites that may be appealing to a community sample, and offers some directions to website developers about ways to maximise the impact and use of these sites. The results underline the importance of tailoring website content, appearance and interactivity options to the target age group and cultural background, and emphasises that there is only a small window of opportunity (perhaps 2 minutes or less) for sites to engage a user with the website's content. Further research should test the generalizability of the current results to other community samples, and to people seeking help for their own AOD problems. It should also examine whether sites that are constructed to meet the criteria established in the current study are actually preferred by site users more than sites that do not, and to see whether the former sites are more effective at engaging participants and promoting behaviour change. Results from such studies can allow us to refine this potentially important component of an effective service mix for people with AOD problems or consumption that puts them at future risk.

## Competing interests

The authors declare that they have no competing interests.

## Authors' contributions

FKL provided input in the the study design and conception, made an original contribution to design of focus group questions and observational data log, took a lead role in data analysis and interpretation. She produced the first full draft of manuscript and in producing the final version incorporating critical comments from authors.

AW made an original contribution to the design of the study, had significant input into the focus group protocol, took a lead role in the acquisition of data, and provided input into the analysis and interpretation of results. AB provided input into the focus group protocol, significant input into the analysis and interpretation of results, and made an original contribution and critical input into drafts of the manuscript. DK took a lead role in the study conception and design, input into the data analysis and interpretation, input into the focus group protocol, and made an original contribution to manuscript and significant input into article revisions. BK, JP, JD, JC and RY each provided significant input into the study conception and design, along with critical and original input into manuscript drafts and revisions. All authors read and approved the final manuscript.

## Pre-publication history

The pre-publication history for this paper can be accessed here:

http://www.biomedcentral.com/1471-2458/11/277/prepub
